# Missed Nursing Care Among Hospital Nurses in the Middle East: A Systematic Literature Review

**DOI:** 10.3390/nursrep16020040

**Published:** 2026-01-26

**Authors:** Bedoor Bader Abdullah, Fathieh Abdullah Abu-Moghli

**Affiliations:** Nursing Department, Faculty of Nursing, The University of Jordan, Amman 11942, Jordan; fathieh@gmail.com

**Keywords:** hospitals, Middle East, missed nursing care, nursing workforce, omitted nursing care, patient safety, unfinished care

## Abstract

**Background/Objectives**: Missed Nursing Care is a global concern that affects nurses’ well-being and patients’ safety. Despite global recognition of Missed Nursing Care, there is limited synthesized evidence that determines its characteristics in a Middle Eastern context. The purpose of the study is to synthesize the existing evidence about the prevalence of Missed Nursing Care among nurses in hospitals, the types of care missed, and reasons for Missed Nursing Care in the Middle East. **Methods**: A systematic literature review is conducted by using a comprehensive search in CINAHL, Scopus, and ScienceDirect databases for studies published between 2020 and 2025 and utilizing the MISSCARE Survey. **Results**: 25 studies met the inclusion criteria. The reported prevalence of Missed Nursing Care ranged between 1.06 and 2.9 out of five, indicating a low to moderate level. Frequent missed care activities included ambulation, hygiene, mouth care, and patient teaching. Contributing factors were staffing shortages, heavy workload, resource limitations, and communication issues. Missed Nursing Care critically affected patients’ outcomes, reduced job satisfaction, and caused moral distress and a higher intent to leave the profession. **Conclusions**: Missed Nursing Care remains a significant, complex challenge in the Middle East. Therefore, understanding this phenomenon in the region is needed. Collaborative efforts among policymakers, administrators, and nursing leaders are essential to implement targeted interventions, supportive policies, and ongoing research to minimize Missed Nursing Care across the Middle East.

## 1. Introduction

Globally, healthcare systems have grown in complexity based on ongoing challenges and continuous medical and technological advancement [1,2,3]. Nursing, within the healthcare system, is considered a central and vital profession, which is expected to provide efficient and safe nursing care for patients [4]. However, nurses may face several clinical, environmental, and administrative challenges within their healthcare facilities, creating situations where nurses may fail to provide all required nursing care for their patients, which negatively influences patients’ safety and overall healthcare quality [4,5].

Missed Nursing Care (MNC) is a global phenomenon, defined as any essential patient care that is omitted, partially delivered, or delayed [3,5,6,7]. This issue is a significant concern due to its direct impact on patient safety, contributing to adverse events like medication errors, nosocomial infections, and patient’s injuries. These events may lead to prolonged hospital stays and increased mortality rate [4,5,8]. MNC also affects the quality of care, patient satisfaction, nurses’ well-being, and increases healthcare costs [8,9,10]. MNC is understood as a result of inadequate staffing, heavy workloads, resource limitations, and miscommunication among the healthcare members, rather than nurses’ negligence [1,4,11,12].

MNC includes common nursing activities such as ambulation, patient education, emotional support, hygiene, mouth care, positioning patients, monitoring, and documentation [7,13,14]. Kalisch and Williams (2009) [13] developed the MNC Survey (MISSCARE Survey) that measured the type and frequency of MNC during the shift. This tool was adopted globally and provided a standardized approach to identify and quantify nursing care omission across different healthcare settings [2,13,14,15].

MNC is a serious global challenge, with significant rates across various healthcare settings. Studies from different countries reported that the prevalence of overall MNC ranges from 6.8% up to 98.1% [4,12,16]. Many factors may contribute to the occurrence of MNC. These factors are categorized by nurse-related factors, patient-related factors, and workplace environmental factors. Nurse-related factors include nurses’ characteristics, such as nurses’ age and work experience [17,18]. Factors such as nurses’ intention to leave, job satisfaction, educational level, and working hours are also found to be significant predictors of MNC [1,12,16]. Patient-related factors such as unexpected rise in patient volume and/or severity of patient’s illness significantly increase the incidence of MNC [4,18,19]. Workplace environmental factors, including inadequate staffing and high nurse–patient ratio, were frequently reported to have led to MNC incidences [1,4,20,21]. Furthermore, limited medical resources (material resources, equipment, and supplies), communication/teamwork issues, non-nursing tasks, and non-supportive leadership are among the consistently reported factors of MNC occurrence [4,11,12,22].

The consequences of MNC affect both nurses and their patients. For nurses, MNC contributes to decreased job satisfaction, increased moral distress, burnout, absenteeism, and a higher intent to leave the profession [3,8,23]. For patients, MNC can result in adverse events such as medication errors, infections, injuries, pressure ulcers, increased morbidity and mortality rates, and prolonged hospital stays. Also, MNC can decrease patient’s satisfaction toward the overall quality of care [4,5,24,25].

Several existing literature reviews and meta-analysis studies have provided comprehensive insight into MNC, globally and in several regions. For example, a systematic review by Gong et al. (2025) [4] conducted a literature review to identify prevalence and reasons for MNC among 14 countries, which reported the prevalence of MNC ranged from 6.8 to 98.1%. Similarly, in Ethiopia, a literature review for 10 studies, conducted among Ethiopian nurses, reported overall prevalence of MNC rate around 55.99%. The literature review highlighted several factors associated with MNC incidence like intention to leave the profession, job satisfaction, and nurses’ educational level [12]. Other literature reviews have investigated the relationship between MNC within several factors, such as Azzellino et al., (2025) [16] who reported the positive relation between MNC and the intention to leave the profession among nurses as a result of job dissatisfaction, lack of organizational support, and staffing issues. Furthermore, other literature reviews focused on specific global events, such as the COVID-19 pandemic, as both Bayram et al. (2024) [26] and Chiappinotto et al. (2023) [27] conducted literature reviews assessing the impact of the pandemic on MNC prevalence and its contributing factors among nurses who were working with COVID-19 patients and non-COVID-19 patients. These literature reviews provided solid differences in MNC incidence during that period and among COVID-19 patients. The findings indicated that the incidents of MNC were significantly higher among nurses who provided care for COVID-19 patients due to increased workload and resource limitations [26,27]. The body of knowledge about MNC, based on the existing literature reviews, mostly covered the prevalence, type, and contributing factors of MNC. These studies were conducted in different areas and within a specific period of time for potentially developing strategic and managerial approaches that may assist in minimizing the incidence of MNC in healthcare facilities.

Despite the growing body of knowledge about MNC, significant gaps remain in the current situation, particularly in the Middle East. Also, the systematic review and meta-analysis that gather evidence about type, causes, and consequences of MNC, for further understanding, highlight a critical gap in exploring the organizational culture in more detail, and initiate effective interventions to reduce the incidence of MNC [4,12]. For the Middle East, while this study in the region provides valuable insights, it also reveals certain variations in the prevalence and contributing factors of MNC, even within the same country [20,21]. These variations can reflect the cultural and organizational contexts within Middle Eastern healthcare facilities [18,22]. Therefore, the global perception about MNC may not reflect the perception of MNC in the region. Apparently, there is a clear need for a systematic review that synthesizes existing evidence about MNC to identify specific regional challenges and opportunities. Therefore, this paper aims to address this critical gap by offering a focused revision of MNC within the Middle Eastern context, thereby contributing to an in-depth understanding that can contribute to the initiation of effective interventions and policies. To meet the aim of this study, there are several research questions that need to be answered:

- What is the reported prevalence of MNC among nurses working in hospitals in the Middle East?
- What are the types of MNC among nurses working in hospitals in the Middle East?
- What are the reasons associated with MNC among nurses in hospitals in the Middle East?
- What are the reported consequences of MNC for patient safety, quality of care, and nurses themselves in Middle Eastern hospital settings?

## 2. Materials and Methods

### 2.1. Design

This study adopted a systematic literature review design to synthesize existing evidence on MNC among nurses working in hospitals in Middle Eastern countries. The design is an appropriate approach to critically identify the existing evidence, identify knowledge gaps, and provide an in-depth overview on MNC among nurses in Middle Eastern hospitals. The review protocol for this study was developed but not registered in PROSPERO or any other accessible registry. Furthermore, this literature review is conducted following the Joanna Briggs Institute (JBI) methodology (Joanna Briggs Institute, Adelaide, Australia) and reported in accordance with the Preferred Reporting Items for Systematic Reviews and Meta-Analyses (PRISMA) reporting guidelines [28,29].

### 2.2. Search Strategy

A comprehensive search was performed in multiple electronic databases including CINAHL (via EBSCOhost; EBSCO Information Serices, Ipswich, MA, USA), Scopus (Elsevier, Amsterdam, The Netherlands), and ScienceDirect (Elsevier, Amsterdam, The Netherlands). These databases were selected to ensure broad coverage of nursing studies. No additional information sources or searching methods were used beyond the three electronic databases, no study registries were searched, no additional online or print sources, and no additional studies or data were sought by contacting authors or experts, as this study focused on peer-reviewed published studies. Furthermore, reference lists of the relevant studies in these databases were not screened to identify additional studies. The decision of relying on electronic databases and excluding additional searching sources and methods was made to ensure focused and consistent searching strategy.

The search strategy included terms related to MNC. The following keywords were used: “missed care”, “missed nursing care”, “nursing care left undone”, “omission”, “omitted nursing care”, “unfinished care”, “nursing care”, “nursing”, “nurses”, “staff nurse”, “hospitals”, and the names of individual Middle Eastern countries. Boolean operators (AND, OR) were applied to combine these terms and broaden the searching strategy effectively. The literature search was conducted once without updates and focused on a timeframe extending between 2020 and October 2025. This selected timeframe provided contemporary evidence about the occurrence of MNC within the period of the COVID-19 pandemic and ensured the most recent studies about MNC among nurses in Middle Eastern hospitals. Therefore, the systematic literature review provided a focused and in-depth revision about MNC among nurses in the Middle East during and after the global crisis. Also, language limitation was applied to English-language publications. No other published search filters were used to ensure in-depth analyses and reporting of the findings.

### 2.3. Selection of the Studies

#### 2.3.1. Inclusion Criteria

- Studies conducted in Middle Eastern countries.
- Studies conducted in the last 5 years (2020–2025).
- Studies conducted in hospitals.
- Studies that included nurses as participants.
- Full-text articles.
- Studies published in the English Language.
- Studies that were peer-reviewed.
- Studies using MISSCARE Survey for data collection.

#### 2.3.2. Exclusion Criteria

Studies were excluded if they had incomplete data, if the full-text article was inaccessible, and if they consisted of review articles, case series, case reports, conference posters, or letters to the editors.

#### 2.3.3. Screening Process

The study selection involved a multi-stage screening process, based on PRISMA 2020 guidelines, to identify eligible articles for the literature review. This multi-stage screening was conducted by two reviewers independently, with discussions of any discrepancies founded between the reviewers. First, in the identification stage, 1038 records were identified by searching in the three electronic databases (ScienceDirect = 350, CINAHL via EBSCOhost = 533, Scopus = 155). Before screening, 860 records were removed, as they were unrelated to the nursing field, leaving 178 records for the next stage.

In the screening stage, title and abstract were screened and 55 records were excluded due to language issues and unavailability of full-text records, leaving 123 records for the next screening. Next, all identified records were imported into reference management software, EndNote (version 21.5; Clarivate Analytics, Philadelphia, PA, USA) [30], for removal of duplicates, leaving 52 records for the next screening and 71 records excluded due to duplication. Next, manual screening of full-text records was conducted for illegibility based on inclusion criteria. In total, 27 records were excluded for the following: qualitative design (*n* = 5), mixed-method study (*n* = 1), pediatric department (*n* = 4), irrelevant population (*n* = 2), not in general department (*n* = 9), and not using MISSCARE tool (*n* = 6).

**Figure 1:**
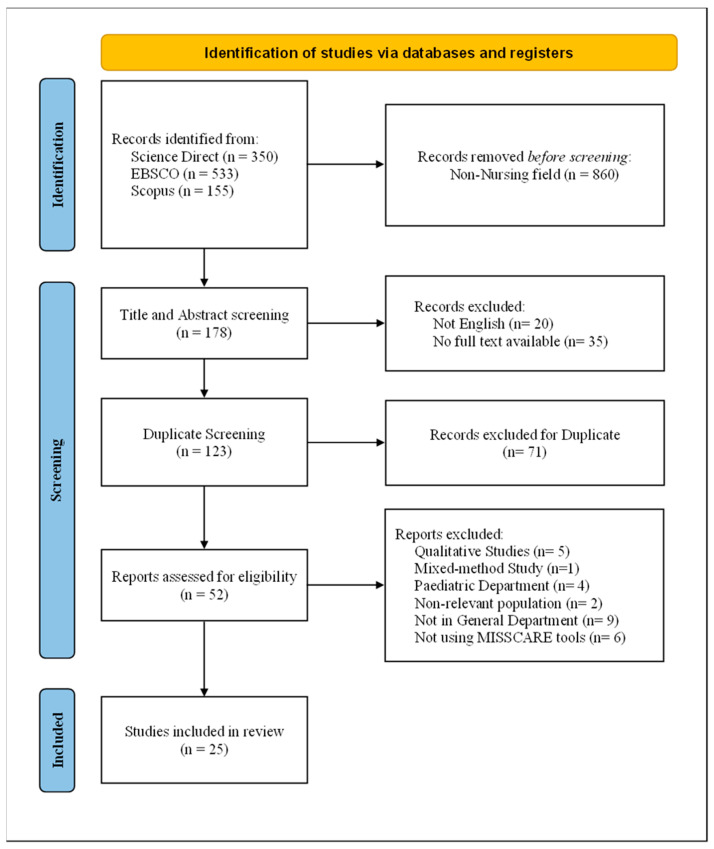
PRISMA 2020 flow diagram illustrating the study selection process.

The reference lists of eligible studies were not examined, and citation tracking was not conducted. Therefore, a total of 25 studies were included for this study. These exclusions at the full-text screening stage may ensure methodological consistency and comparability across the findings of the included studies. Furthermore, limiting the inclusions to studies using MISSCARE Survey may minimize the potential heterogeneity of the findings among the studies, thereby allowing for a strengthening of the interpretation of the findings based on the research questions of this systematic literature review. The screening process is summarized and presented using PRISMA flow diagram [29] (see Figure 1).

### 2.4. Data Extraction

A structured data extraction form was used to collect key information from each included study. Data was extracted using a standardized form from the JBI data-extraction tool described by Aromataris and Munn (2020) [28]. The data extraction was performed by two researchers independently from each included study. This process ensured accuracy during data extraction and minimized potential bias. Extracted data included: author(s), year of publication, country, study design, hospital setting, population and sample size, measures of MNC, key findings, and quality appraisal outcomes.

Next, a structured narrative was synthesized to ensure consistency and comparability in the findings. The two researchers independently extracted data into a table and synthesized the findings according to the research questions. The synthesized findings were structured according to the following: the reported prevalence of MNC, types of the most frequent MNC, reasons of MNC, and the consequences of MNC. Then, the two researchers compared their reported synthesized findings to identify similarities and differences. Any discrepancies between the two studies were resolved through discussion.

### 2.5. Quality Appraisal and Risk of Bias

The methodological quality of the included studies was evaluated using the JBI Critical Appraisal Checklist to analyze the eligible studies [28]. The conduction of the quality appraisal was performed by two researchers independently, with discussion of any discrepancies found between the reviewers. The results of this quality appraisal were reported to identify the strength of the evidence base for each included study, highlighting potential limitations; but, it was not used to exclude any included studies, as the primary aim of this literature review is to synthesize a literature review from the existing evidence.

### 2.6. Data Synthesis

The results were narratively synthesized to summarize and integrate the findings across the included studies and to provide an evidenced-based descriptive overview about MNC among nurses in Middle Eastern hospitals. Data were organized into themes: the prevalence of MNC, the most common types of MNC, the most common reason of MNC, contributing factors, and consequences. The similarities and differences across the studies were identified, and gaps in the included studies were highlighted.

## 3. Results

### 3.1. Overview of Included Studies

The identification and selection of the studies for this literature review was performed through a multi-stage screening process, which was illustrated using PRISMA guidelines and a flow diagram [29] (Figure 1). Following the stages, which started from title and abstract screening to full-text assessment for eligibility, a total of 25 studies were included for data extraction and synthesis for this literature review.

The included studies for this literature review are [1,3,9,10,11,17,18,19,20,21,22,31,32,33,34,35,36,37,38,39,40,41,42,43,44]. A descriptive summary of the 25 included studies was integrated by the structured data extraction form [28] (Table 1).

As summarized in Table 1, the included studies were conducted in various countries within the Middle East, such as Türkiye (9 studies), Iran (8 studies), Jordan (3 studies), Kingdom of Saudi Arabia (KSA) (3 studies), and Egypt (2 studies). All the included studies employed a quantitative approach with cross-sectional or descriptive correlational design. Moreover, all these included studies were conducted in between one to twenty-five healthcare facilities across the Middle East. These healthcare facilities varied based on affiliation: Public, Private, Teaching, or University Hospitals. Regarding the targeted population, nurses were the targeted population, and the sample size of the targeted population ranged from 183 to 1310 nurses. Furthermore, all the included studies were primarily using MISSCARE Survey as the data collection tool, with several studies (*n* = 20) using additional data collection tools to examine nursing, organizational, or environmental factors (e.g., moral sensitivity, professional commitment, fatigue, leadership, and work environment).

Based on the key findings, in Table 1, the overall MNC score in most of these studies was reported at a low to moderate level. The most common types of MNC reported in the included studies were ambulation, hygiene, planning, and patient education. Several reasons for MNC were highlighted in the included studies: staff shortage, workload, nurse–patient ratio, shift type, high admission and discharge rate, human and material resource issues, and patient acuity. However, most of the included studies did not explicitly report the consequences of MNC. Few studies reported job satisfaction, intent to leave the profession, and reduced quality of care. Regarding the quality appraisal of the included studies, all studies met the minimum quality threshold and were, therefore, included in the synthesis. The results of the quality appraisal were used to support the interpretation of findings rather than to exclude studies based on their quality score.

### 3.2. Thematic Synthesis of MNC

#### 3.2.1. Prevalence of MNC

The included studies reflected that the prevalence of MNC ranged between 1.06 and 2.9 out of 5, indicating a low to moderate level of MNC in the Middle East [3,10,36,38]. In Turkey, a study reported an overall mean score of MNC around 1.06 out of 5, which is the lowest score in the region [35]. Another study conducted in 13 healthcare facilities reported a mean score of MNC around 1.63 out of 5 [10]. Furthermore, one study conducted in 10 healthcare facilities reported a higher mean score, around 2.9 out of 5, which is the highest score in the region [19]. Studies in Iran reported a mean score of overall MNC around 1.99 out of 5 in two University hospitals [44]. Another study reported a score of 2.57 out of 5 in eight healthcare facilities [36]. In Egypt, a reported prevalence of MNC rate was around 2.26 out of 5 [11]. The other included studies reported the mean score for each nursing activity rather than including the overall mean score in their results. This includes studies in Jordan and KSA. However, the studies in KSA reported that the mean score of overall MNC is considered to be at a moderate level [20,21]. The variation in the reported prevalence of MNC was identified based on:

- Hospital type: Public hospitals found to have a higher mean score for MNC compared to private and university-affiliated hospitals [10,36].
- Settings: Intensive care units and surgical departments have a lower mean score compared to medical departments [9,22,39].
- Nurses’ characteristics: Male nurses showed a higher mean score compared to female nurses [3,18,43]. Also, educational level and working experience were identified as predictors for MNC [3,11,39].
- Nurse-to-Patient Ratios: A significant positive relationship exists between MNC and the nurse-to-patient ratio [11,17,19,36].
- Workplace Characteristics: Heavy admissions/discharges and unexpected emergencies due to patient’s acuity were identified as significant predictors of MNC [17,21,34,39].

#### 3.2.2. Most Common Types of MNC

The included studies have also reflected similarities between the types of MNC found in the Middle East, and the types reported in the rest of the region. These types of MNC activities included patient ambulation, hygiene, mouth care, set up meals, feeding the patients, and nurses’ attendance in interdisciplinary conferences [20,36,38]. Yet, there are more common types of MNC that make distinctions among Middle Eastern countries. In Turkey, activities like administration of PRN medication and emotional support for patients and family were found to be the most common types [10,19]. In both Iran and Egypt, discharge planning and patient teaching were found to be the most commonly missed [11,36].

#### 3.2.3. Most Common Reasons for MNC

The often perceived reasons for MNC in the Middle East are staffing shortage, heavy admission and discharge, unexpected high patient volume or acuity, communication factors, material resources, and teamwork. These reasons were found to be the most common in the whole Middle East region, based on the included studies [3,17,38]. However, other reasons were found to be distinctively common in certain countries. In Turkey and Saudi Arabia, inadequate assistive personnel were found to be among the common reasons of MNC [21,34]. Also, in Jordan, human resource was found to be common reason as perceived by Jordanian nurses [3].

#### 3.2.4. Contributing Factors to MNC

- Nurse-Related Factors: Several contributing factors based on individual nurse characteristics, such as age, gender, working experience, educational level, and type of department, were significantly associated with MNC [10,17,19,38]. Moreover, factors like intentions to leave, job satisfaction, educational level, and working hours were also significant predictors for MNC [3,11,20,34]. Furthermore, occupational fatigue, based on the included studies, is reported to cause physical, cognitive, and emotional exhaustion, which increased the risk of missed care [21]. On the other hand, the reviewed studies reported that professional commitment, moral competence, shared leadership, and transitional shock were negatively correlated with MNC, leading to less MNC incidents [9,35,42,44].
- Organizational/Systemic Factors: Several challenges within the healthcare organization were considered as contributing factors. These factors are inadequate staffing levels, high nurse-to-patient ratios, workload, shifts, resource limitations (including medical resources), non-nurse-supportive leadership, communication issues (including teamwork issues), and non-nursing tasks [3,9,17,36].
- Environmental Factors: Certain external factors were reported to influence the MNC incidence, such as pandemic crises. Studies highlighted the impact of the COVID-19 pandemic, which led to increased workload and patients’ admission and discharge rate, which significantly exacerbated the prevalence of MNC [22,32,39]. Also, the type of workplace environment and perceived organizational support were identified as significant determinants [1,32].

#### 3.2.5. Reported Consequences of MNC

Almost all the included studies highlighted the factors that increase the incidences of MNC. However, Al-Faouri et al. (2020) [3] and Dirgar et al. (2024) [41] highlighted the consequences of MNC on the nursing profession, which are decreased job satisfaction level, increased moral distress, absenteeism, and a higher intent to leave the profession.

## 4. Discussion

### 4.1. The Prevalence of MNC in Middle East

The overall prevalence of MNC in Middle Eastern countries ranged from low to moderate level. These variations in the overall prevalence of MNC are observed across different studies in different regions. In European countries, the overall prevalence of MNC ranges from 75% to 88%, indicating moderate to high MNC score [6]. In US, the reported MNC ranged from low to moderate level [14]; in New Jersey, the MNC level ranged from 10% to 27%, indicating low level [36]. Globally, the MNC level was reported to be between 10% and 50%, which indicated low to moderate level. These findings in different regions indicated that MNC is not limited to cultural or economic differences [6,8,45]. Moreover, when considering these international findings, the Middle Eastern rates appear to fall within the lower to moderate range, when compared with the global spectrum. Yet, these comparisons require further analysis due to the variation in methodological and data collection strategies among the studies. Furthermore, studies in the Middle East mostly reflect the countries with high research activities toward this phenomenon, as most of these studies reflect MNC in Iran and Türkiye.

### 4.2. Common Types of MNC in Middle East

Studies in the Middle East region reported that the most common type of MNC were basic nursing care, such as ambulation, hygiene, mouth care, feeding patients, emotional support, and attendance conferences. There were minor regional distinctions such as administering PRN medication and emotional support in Turkey, and discharge planning and patient teaching in Iran and Egypt [11,19,34,36]. In American and European regions, such as US, Italy, and Denmark, studies reported similar basic nursing care that been missed among nurses in the Middle East [25,45,46]. Furthermore, a study conducted in South Korea reported similar findings to the Middle East and European region [47]. These similarities across different geographical regions indicate that nurses provided nursing care based on priorities [48]. Nurses focused on providing immediate life-saving care for their patients, resulting in potential missing of other non-immediate nursing care, even if these were considered as basic nursing care [4,14,47,49].

### 4.3. Common Reasons for MNC Perceived by Nurses in Middle East

The most reported reasons for MNC were staff shortage, heavy admission and discharge rate, workload, limited resources, patient acuity, communication, and teamwork [10,17,23,36]. These findings have been mostly reported in Middle Eastern and non-Middle Eastern regions [4,17,47,50]. The similarity of these reasons in different regions highlighted the nature of MNC, where this phenomenon reported higher levels of staffing shortage, limited resources, workload imbalance, and teamwork issues in a healthcare facility, regardless of the country and the culture [4,6,12,16].

### 4.4. Contribution Factors to MNC in Middle East

The contributing factors to MNC are categorized based on the nurses’ characteristics, organizational/systemic, and environmental factors. Each plays a vital role causing MNC.

Regarding the nurses’ characteristics factors, these include age, working experience, educational level, and psychological well-being [17,43,51,52]. Several studies reported nurses in their 30s commit less MNC compared to under 30s. However, Erdat et al. (2024) [35] found that newly graduated nurses have low MNC rate. These contradictions reflected the amount of responsibility that nurses of different ages have. For new graduate nurses, the responsibility and tasks were minimal compared to older nurses. Also, by age 30s, nurses are more competent and skilled enough to minimize MNC rates [19,34,35]. Regarding the educational level and working experience, nurses with higher educational levels and greater working experience have effective time-management skills, and professional commitment, which indicate their potential to reduce the incidence of MNC [1,31,42,44]. On the other hand, one study addressed that nurses with higher experience may tend to commit MNC more. This could be due to the high administrative tasks and leadership duties that have been held by highly experienced nurses [3,25]. For the psychological well-being, such as moral sensitivity, professional commitment, and occupational fatigue, these were significantly associated with a high incidence of MNC [23,33,42,51]. These factors may drive nurses toward mental fatigue and moral distress, leading to physical, cognitive, and emotional exhaustion, which results in a failure to provide adequate nursing care for the patients [21,51,52].

Organizational/Systemic factors, including hospital type, department type, staffing, nurse-to-patient ratios, workload, leadership style, and non-nursing tasks, were found to be associated with MNC rate [11,19,50]. Studies found that public hospitals have shown to have high MNC rate compared to private and university-affiliated hospitals [19,38,49]. This variation could be due to several reasons, such as public hospitals often serving a larger and more diverse patient population, potentially operating under limited resources, and facing higher patient loads and acuity levels, compared to other hospitals [5,10,38,45]. For the department type, studies that conducted in multiple departments reported that intensive care units and surgical departments tend to have lower mean scores for MNC compared to medical departments [4,12,49]. This may be related to the nature of these departments. ICUs and surgical settings often have stricter staffing ratios, closer monitoring, and a higher priority of nursing care to prevent negative patient outcomes [11,24]. The studies also reported a significant relation between MNC rate and the workload, which occurred due to staffing shortage, high nurse-to-patient ratio, and overload with non-nursing tasks [11,12]. This challenge was found in Middle Eastern studies and non-Middle Eastern studies, due to its significant association with high MNC rate [3,6,50].

Regarding the environmental factors, the literature stressed the effect of external factors, such as pandemics (e.g., the COVID-19 crisis), which can exacerbate workload and patient admission/discharge rates [22,32,39]. This matter was discussed in non-Middle Easter studies, highlighting the complexity of the crisis, where nurses are exposed to more workload and less resources, leading to high MNC rate [26,27]. Moreover, workplace environment and perceived organizational support, are identified as determinants of MNC [1,9]. Lake et al. (2020) [50] and Cho and Steege (2025) [52] highlighted the effect of improved workplace environment and nurse-supportive leadership style on the MNC rate. This improvement may allow effective communication and teamwork among healthcare providers, as well as providing psychological safety for the nurses, leading to minimizing the incidence of MNC [42,45].

### 4.5. Consequences of MNC

The MNC significantly impacts both nurses’ and patients’ outcomes and the well-being of nurses, leading to negative consequences within healthcare systems.

#### 4.5.1. Nurse Outcomes

MNC creates severe professional, psychological consequences on nurses [16]. Nurses who frequently commit MNC are prone to report decreased job satisfaction level, increased moral distress, and height stress levels [3,20,21]. Moral distress occurs when nurses are aware of the nursing care and intervention that must be provided for their patients, but they end up missed or delayed due to clinical and administrational constraints or insufficient resources [37,40,42]. This continuous struggle to meet patients’ needs can lead to low job satisfaction and burnout, characterized by physical, cognitive, and emotional exhaustion [3,11]. Moreover, studies show that occupational fatigue directly increases the risk of MNC, leading to further exacerbation [21]. These factors contribute to absenteeism and a high risk of intent to leave the nursing profession [16,41].

#### 4.5.2. Patient Outcomes

There is a direct and critical link between MNC and an increase in adverse events for patients [12]. When basic nursing care is missed, delayed, or partially delivered, patients may become at a higher risk of experiencing medication errors, hospital-acquired infections, injuries, and the development of pressure ulcers [21,24]. These omissions primarily deteriorate patient safety and can lead to preventable health complications [4,5,24]. The occurrence of these adverse events directly contributes to several negative patient outcomes, including prolonged hospital stays, increased rates of morbidity, and, in severe cases, higher mortality rates [12,21,45]. Beyond these clinical outcomes, MNC also negatively impacts patient satisfaction and the overall quality of care provided in healthcare facilities [5,45].

### 4.6. Strength and Limitations

#### 4.6.1. Strengths

This systematic literature review applied a consistent methodological approach by following the JBI methodology and reported in accordance with PRISMA reporting guidelines. This approach provided well structured searching strategies through health and nursing databases, which helped to identify the relevant literature and reduce the likelihood of missing relevant studies. Moreover, the targeted timeframe of the systematic literature review ensured the inclusion of the most recent evidence, as well as the evidence of a specific global event (COVID-19 pandemic), to provide an in-depth understanding of MNC.

Furthermore, including studies that mainly used the MISSCARE Survey enhanced the comparability of the findings and identification of the variations between the studies. This may provide an accurate assessment of the prevalence, types, and reasons of MNC in the Middle East, making the analysis more reliable. Also, the literature review focused on analyzing the studies conducted in the Middle East, which are yet to be studied in global health literature. This allows for an in-depth understanding of MNC within a unique cultural context and contributing to focused insights into that region.

#### 4.6.2. Limitations

Several limitations should be acknowledged. First, limiting the inclusion criteria for the included studies to the English language and limited timeframe may exclude relevant studies. Second, there is a potential of publication bias, as the studies with statistically positive findings are more likely to be published than those with no significant results. This may lead to mis-conceptualized perception about the prevalence or impact of MNC. Third, the searching strategy was limited to studies conducted in hospitals and among registered nurses. This means that MNC in other healthcare settings (out-patient clinics and homecare) or among other care providers (nursing assistants) were not included, leading to an incomplete picture of MNC in the Middle East. Finally, although the inclusion criteria aimed for consistency, including the studies that mainly used the MISSCARE Survey may exclude relative findings.

### 4.7. Implications

#### 4.7.1. Implications for Nursing Practice

This systematic literature review demonstrates a low to moderate MNC rate in Middle Eastern healthcare facilities. The most common types of MNC were classified as basic nursing activities, such as ambulation, hygiene, feeding the patients, and emotional support. Therefore, in-service education and continuous professional development programs are needed to increase nurses’ awareness about MNC and motivate nurses to take proper actions to minimize MNC.

Furthermore, the reasons for MNC occurrence were related to staff shortage, limited resources, workload, communication, and teamwork issues. Thus, including nursing managers and other healthcare leaders in professional development programs is necessary to enhance awareness of MNC and the importance of providing effective staffing, resource management, workload management to minimize non-nursing tasks, and enhance communication and teamwork among the healthcare providers by empowering interdisciplinary collaboration, assuming these professional development programs may minimize the rate of MNC in Middle Eastern healthcare facilities.

#### 4.7.2. Implications for Healthcare Policy and Management

This systematic literature review highlighted organizational factors, such as staff shortage, resource limitation, and non-supportive leadership, as contributors to MNC in Middle Eastern healthcare facilities. Therefore, prioritizing leadership development programs is recommended to promote a nurse-supportive leadership style, promote effective communication and teamwork, and create a positive organizational culture that prioritizes patient safety and nurses’ well-being.

In addition, there is a variability in MNC prevalence and types based on the hospital type and clinical settings, indicating the need for regular assessments of the workplace environment to identify the causes of MNC within specific environmental contexts. Also, ongoing monitoring and evaluation of MNC rates and contributing factors are needed to guide policy adjustments and assess the effectiveness of managerial interventions, particularly during exceptional periods, such as a pandemic crisis.

#### 4.7.3. Implications for Nursing Research

This systematic literature review revealed that most of the studies about MNC in the Middle East were conducted in Türkiye and Iran, with limited representation about MNC from other countries in the region. Therefore, future research should expand to the unrepresentative countries in the Middle East to provide a more comprehensive understanding of MNC prevalence, type, reasons, and consequences.

Moreover, almost all studies were cross-sectional study designs, highlighting methodological gaps in nursing research. Thus, qualitative studies are needed to explore nurses’ lived experience of MNC. Also, longitudinal research designs are required to examine the long-term impacts of MNC on nurses’ and patients’ outcomes, and overall healthcare service quality. Furthermore, there is a demand to conduct an experimental and quasi-experimental study to evaluate the effectiveness of clinical and administrative interventions that aim to reduce MNC in Middle Eastern healthcare facilities.

## 5. Conclusions

MNC remains a significant challenge across hospitals in the Middle East, with significant negative consequences for both patients and nurses. While MNC is a globally recognized issue, this review highlights the unique cultural, and organizational factors in the Middle Eastern region that may describe its prevalence, type, causes, and outcome. The overall MNC prevalence in the region ranges from low to moderate, with considerable variations related to hospital type, clinical setting, and nurse-to-patient ratios. Types of MNC that commonly occur are mainly fundamental nursing care, such as ambulation and hygiene, as well as patient’s education and emotional support. The consequences are significantly serious, such as increased adverse events, prolonged hospital stay, increased risk of morality, nurse burnout, moral distress, and intention to leave.

The validity of this literature review lies in its systematic and regional focused approach, which includes relevant studies that use the MISSCARE Survey and provides a thematic synthesis based on evidence. However, limitations such as methodological heterogeneity, publication bias, and covering only cross-sectional studies must be acknowledged. Therefore, addressing MNC requires a comprehensive approach that prioritizes adequate staffing, efficient resources, effective teamwork, and nurse well-being. At the policy level, implementing effective staffing policies and encouraging nurse-supportive leadership are essential. Future research is needed to include qualitative and longitudinal studies exploring organizational culture and interventions.

## Figures and Tables

**Table 1:** Characteristics of the included studies (=25 studies).

Authors/Year/Country:	Study Design:	Setting:	Population/Sample Size:	Tools for Data Collection	Key Findings:	* Quality Appraisal: (Y/N/U/NA)
1. Hendy et al., (2024) [1] Egypt	Cross-sectional	Total 7 Hospitals	Nurses (813)	MISSCARE Survey and 2 more tools	Moderate MNC score. Reasons: resources; communication. High MNC is associated with low professional commitment and work environment.	8/0/0/0
2. Al-Faouri et al., (2020) [3] Jordan	Cross-sectional	Total 3 Public, Private, and University Hospitals	Nurses (300)	MISSCARE Survey	Low–moderate MNC score. Type: ambulation; teaching patients. Reasons: high nurse–patient ratio; staff shortage. High MNC associated with low job satisfaction and high intention to leave.	8/0/0/0
3. Ghanem Atalla et al., (2025) [9] KSA	Cross-sectional	University Hospital	Nurses (340)	MISSCARE Survey and 1 more tool	Moderate MNC score. Type: secondary care. Male nurses, <30 years old, and married experienced high shared leadership and low MNC.	8/0/0/0
4. Cimen et al., (2021) [10] Türkiye	Cross-sectional	Total 13 Public and University Hospitals	Nurses (550)	MISSCARE Survey	Low–moderate MNC score. Type: ambulation; positioning patients. Reasons: staff shortage; workload. High MNC in Public Hospitals than University due to workload.	6/1/1/0
5. Hammad et al., (2021) [11] Egypt	Cross-sectional	Teaching Hospital	Nurses (533)	MISSCARE Survey and 1 more tool	Moderate MNC score. Type: planning; teaching patients. Reasons: staff shortage; high admission; non-nursing tasks. High MNC associated with high non-nursing tasks and low job satisfaction.	7/0/1/0
6. Khajoei et al., (2025) [17] Iran	Cross-sectional	Total 2 Hospitals	Nurses (189)	MISSCARE Survey and 1 more tool	Moderate MNC score. Type: Toileting; feeding patients. Reasons: staff shortage; non-nursing tasks. MNC is associated with nurses’ age and experience.	8/0/0/0
7. Eskin Bacaksiz et al., (2020) [18] Türkiye	Cross-sectional	Total 25 Private Hospitals	Nurses (897)	MISSCARE Survey and 3 more tools	Low–moderate MNC score. Type: Ambulation. Reasons: staff shortage; workload. High MNC associate with gender, weekly working hours, and high powerless score.	8/0/0/0
8. Taskiran Eskici et al., (2022) [19] Türkiye	Cross-sectional	Total 10 Public, Private, University Hospitals	Nurses (1310)	MISSCARE Survey and 1 more tool	High MNC score. Type: positioning patients. Reasons: staff shortage; teamwork. MNC significantly affected by nurses’ experience, nurse–patient ratio, and teamwork.	8/0/0/0
9. Alshammari, Alshammari, and Baghdadi, (2025) [20] KSA	Cross-sectional	Total 7 Public Hospitals	Nurses (366)	MISSCARE Survey and 1 more tool	Moderate MNC score. Type: oral care; bathing; feeding patients. Reasons: staff shortage; high nurse–patient ratio. High MNC associated with low job satisfaction.	7/1/0/0
10. Alshammari et al., (2025) [21] KSA	Cross-sectional	Total 5 Hospitals	Nurses (183)	MISSCARE Survey and 1 more tool	Moderate MNC score. Type: patients hygiene; ambulation. Reasons: staff shortage; high admission and discharge. High MNC associated with fatigue and low inter-shift recovery.	7/1/0/0
11. Alfuqaha et al., (2023) [22] Jordan	Cross-sectional	Tertiary Hospital	Total nurses: (260) pre-COVID and during COVID	MISSCARE Survey	MNC score increased during pandemic. Type: ambulation. Reasons: staff shortage; workload. High MNC associated with workload, job satisfaction, absence, and intention to leave.	8/0/0/0
12. Tiryaki Sen et al., (2025) [31] Türkiye	Descriptive Correlational	Public, Private, and Education and research Hospitals.	Nurses (514)	MISSCARE Survey and 2 more tools	Low–moderate MNC score. Type: planning. Reasons: high–nurse-patient ratio; workload. High MNC associated with low conscientious intelligence.	7/0/1/0
13. Khrais et al., (2023) [32] Jordan	Cross-sectional	Total 8 Public, Private, and Teaching Hospitals	Nurses (536)	MISSCARE Survey and 2 more tools	Reasons: communication; resources issues. Under the impact of COVID-19, High MNC is associated with low accountability and perceived organizational support.	8/0/0/0
14. Taskiran Eskici et al., (2025) [33] Türkiye	Descriptive Correlational	Public and Private Hospitals	Nurses (640)	MISSCARE Survey and 2 more tools	Moderate MNC score. Type: emotional support; planning. Reasons: workload; staff shortage. High MNC is associated with low professional values and low moral sensitivity.	8/0/0/0
15. Bakar and Canlı, (2024) [34] Türkiye	Cross-sectional	Training and Research Hospital	Nurses (400)	MISSCARE Survey and 1 more tool	Low–moderate MNC score. Type: ambulation; positioning patients. Reasons: staff shortage; patient acuity. High MNC associated with high workload.	6/1/1/0
16. Erdat et al., (2024) [35] Türkiye	Descriptive Correlational	Total 4 Hospitals	New graduate nurses (201)	MISSCARE Survey and 2 more tools	Moderate MNC score. Low holistic competence associated with high MNC. Low transitional shock associated with high MNC.	8/0/0/0
17. Chegini et al., (2020) [36] Iran	Cross-sectional	Total 8 Public and Private Hospitals	Nurses (215)	MISSCARE Survey	Moderate MNC score. Type: planning; teaching; emotional support. Reasons: staffing: limited resources: communication. High MNC associated with gender (male), and age.	8/0/0/0
18. Ahansaz et al., (2024) [37] Iran	Cross-sectional	Medical Education Center	Nurses (202)	MISSCARE Survey and 1 more tool	Moderate overall MNC score. Reasons: staff shortage; workload; shift type. High MNC is associated with low moral sensitivity.	8/0/0/0
19. Dursun Ergezen et al., (2023) [38] Türkiye	Cross-sectional	Total 7 Public Hospitals	Nurses (477)	MISSCARE Survey	Moderate MNC score. Type: emotional support; bathing; ambulation. Reasons: staff shortage; nurse–patient ratio; admission rate. High MNC associated with unit type, experience, intention to leave.	8/0/0/0
20. Mehrabian et al., (2023) [39] Iran	Cross-sectional	Total 7 Educational and Medical Centers	Nurses (326)	MISSCARE Survey and 1 more tool	High MNC score during pandemic. Type: Attending conferences; emotional support; teaching. Reasons: human resources; staff shortage; workload. High MNC is associated with occupational stress and rotation shifts.	8/0/0/0
21. Fouladi et al., (2024) [40] Iran	Cross-sectional	Affiliated University Medical Centers	Nurses (345)	MISSCARE Survey and 2 more tools	Low–moderate MNC score. MNC score is associated with nurses’ characteristics (age, experience, and education) and overtime. High MNC is associated with low moral sensitivity and high workload.	8/0/0/0
22. Dirgar et al., (2024) [41] Türkiye	Descriptive Correlational, Observational	Total 2 Public Hospitals	Nurses (299)	MISSCARE Survey and 1 more tool	Moderate MNS score. Type: Ambulation. Reasons: material shortage; staff shortage; paperwork. High MNC is associated with high presenteeism and reduced care quality.	7/0/1/0
23. Nazari et al., (2024) [42] Iran	Descriptive Correlational	Total 6 University Hospitals	Nurses (200)	MISSCARE Survey and 1 more tool	Moderate MNC score. Type: planning; teaching patients. Reason: workload. High MNC is associated with low moral competence.	7/0/1/0
24. Ahmadzadeh-Zeidi et al., (2024a) [43] Iran	Cross-sectional	Total 2 University Hospitals	Nurses (270)	MISSCARE Survey and 1 more tool	Moderate overall MNC score. Types: planning; teaching patients. Reasons: work-family conflict; staff shortage. High MNC associated with work–family conflict.	7/0/1/0
25. Ahmadzadeh-Zeidi et al., (2024b) [44] Iran	Cross-sectional	Total 2 University Hospitals	Nurses (270)	MISSCARE Survey and 1 more tool	Moderate overall MNC score. Type: planning; emotional support. Reasons: staff shortage; workload. High MNC is associated with low professional commitment.	7/1/0/0

* The first number indicates the number of “Yes” answers; the second number indicates the number of “No” answers; the third number indicates the number of “Unclear” answers; and the fourth number indicates the number of “Not Applicable”.

## Data Availability

Data sharing is not applicable to this systematic literature review as no datasets were generated or analyzed during this systematic literature review.

## Public Involvement Statement

No public involvement in any aspect of this research.

## Guidelines and Standards Statement

This study followed PRISMA 2020 reporting guidelines.
